# The fast-track outpatient clinic significantly decreases hospitalisation rates among polymyalgia rheumatica patients

**DOI:** 10.1186/s41927-021-00210-6

**Published:** 2021-10-05

**Authors:** Stavros Chrysidis, Philip Rask Lage-Hansen, Nikoletta Svendsen, Andreas P. Diamantopoulos

**Affiliations:** 1 Department of Rheumatology, South-west Jutland Hospital, Finsensgade 35, 6700 Esbjerg, Denmark; 2 OPEN, Odense Patient data Explorative Network, Odense University Hospital, Odense, Denmark; 3 Department of Rheumatology, Martina Hansens Hospital, Baerum, Norway

**Keywords:** Polymyalgia rheumatica, Health economics, Disease management, General practice

## Abstract

**Objectives:**

This study aimed to investigate the hospitalisation rates and the reasons for hospitalisation in patients with polymyalgia rheumatica (PMR). Furthermore, it aimed to clarify the impact of a newly established Fast Track Clinic (FTC) approach on hospitalisation rates in connection with PMR diagnosis.

**Methods:**

Patients diagnosed with PMR at South-West Jutland Hospital, Denmark, between 2013 and 2018 were included retrospectively. Only patients fulfilling the 2012 EULAR/ACR classification criteria were included in our cohort. An FTC for patients suspected of having PMR was established in the rheumatologic department of South-West Jutland Hospital in January 2018.

**Results:**

Over 6 years (2013 to 2017), 254 patients were diagnosed with PMR, 56 of them while hospitalised. Hospitalised patients were more likely to have a higher initial CRP mean ± standard deviation (SD) 99.53 ± 59.36 vs 45.82 ± 36.96 mg/lt (*p* <  0.0001) and a shorter duration of symptoms (*p* = 0.0018). After implementing the FTC, a significant decrease in hospitalisation rates (from 20.4% to 3,5%) and inpatient days of care (mean ± SD 4.15 ± 3.1 vs 1 ± 0) were observed. No differences between the two groups were observed regarding clinical symptoms, laboratory values and initial prednisolone dose.

**Conclusion:**

A substantial number of patients are hospitalised in connection with the PMR diagnosis. The FTC approach can decrease the hospitalisation rates significantly among these patients.

**Trial registration:**

Retrospectively registered.

**Supplementary Information:**

The online version contains supplementary material available at 10.1186/s41927-021-00210-6.

## Background

Polymyalgia rheumatica (PMR) is characterised by shoulder and hip girdle pain accompanied by systemic inflammation [1]. The incidence increases with age, and PMR is rarely seen before the age of 50 years. Populations of Scandinavian ancestry are at higher risk of developing PMR [2].

The vast majority of PMR patients are diagnosed and managed in general practice [3–5]. Diagnosing PMR can be challenging as many conditions can mimic the disease [6]. The variety of symptoms may lead to hospitalisation to rule out other serious diseases [7]. In a study from the United Kingdom [8], a majority of general practitioners (GPs) reported that they considered several other conditions (i.e., GCA, infection, malignancy) as the cause of symptoms in their differential diagnosis. Treatment cannot be used in a diagnostic approach as the patient’s immediate response to low dose (15-20 mg) prednisolone daily is not always achieved within a few days/weeks after treatment initiation [9, 10].

Very limited data on hospitalisation rates regarding PMR diagnostics exist [7, 11, 12] and, to our knowledge, the hospitalisation rates in patients diagnosed with PMR have only been mentioned in one short communication [12]. Furthermore, in a symposium review [13], a researcher group described the implementation of a rapid-access PMR clinic, where patients with PMR suspicion were accessed within 2–3 weeks (normal referral time 8–10 weeks) [13]. Fast Track Clinics (FTC) focusing on GCA patients have shown an improvement in patient outcomes and a reduction in medical expenses [14, 15].

Thus, the aims of this study were to investigate the hospitalisation rates and the initial reasons for hospitalisation in patients with PMR. Furthermore, to evaluate the impact of a newly established FTC approach on the hospitalisation rates related to PMR diagnosis.

## Methods

This retrospective observational cohort study was conducted at the Department of Internal Medicine and Rheumatology at South-West Jutland Hospital in Esbjerg (catchment area of 270,000 inhabitants). The South Denmark Region Committees on Health Research Ethics reviewed the study and concluded that the study does not fall under the scope of the Medical Research Involving Human Subjects Act and that written informed consent was not mandatory.

In Denmark, all GPs can refer a patient for further investigation to the secondary health care, as a part of The Danish healthcare system is based on the principles of free and equal access to healthcare for all citizens. PMR patients can be treated in the primary sector, and no specific criteria for which PMR suspected patients should directly be referred to a rheumatologist exist in Denmark. The decision is up to the single GP.

In South-West Jutland Hospital, as in the rest of the country, the GPs can refer a patient to the medical emergency department, where the physicians decide whether the patients will be immediately discharged or be hospitalised for further examinations and treatment. In the South-West Jutland Hospital medical emergency department, the admitted patients can either have a short stay of up to 6–8 h and then immediately discharged or hospitalised. The patients can stay up to 3 days in the medical emergency department or be forwarded to one of the seven internal medicine specialities. The rheumatologists serve primarily the outpatient clinic and the inpatient ward with approximately five beds. During the study period, this arrangement remained unchanged.

Patient identification: All patients registered with a diagnosis of PMR in the Department of Rheumatology in South-West Jutland Hospital from April 2013 to December 2018 were identified using the electronic medical record system (Cambio COSMIC) introduced in our hospital in April 2013. Each patient’s medical record was carefully evaluated. The following baseline data were registered: Age, gender, duration of symptoms, presence of bilateral shoulder pain, inflammatory markers (cut-off for elevated inflammatory markers C-reactive protein-CRP > 10 mg/lt and/or Erythrocyte sedimentation rate-ESR > 30 mm/hr), morning stiffness more than 45 min, presence of rheumatoid factor (RF) and/or Anti-cyclic citrullinated peptide (Anti-CCP), hip pain/decreased range of motion, other joint involvement, fulfilment the 2012 EULAR/ACR classification criteria [16] for PMR, initial prednisone dose, imaging examinations. For hospitalised patients, the initial reason for hospitalisation, the number of inpatient days of care, the initial treatment, and the use of imagining modalities were also recorded.

At baseline, patients with a concomitant rheumatic disease or coexisting GCA symptoms/findings (headache, jaw claudication, tender/thickened temporal arteries, and vision disturbances) were excluded. Due to the absence of accessibility of all patients diagnosed before 2013, it was not possible to estimate the annual incidences of newly diagnosed patients for the years before Cosmic establishment; therefore, patients diagnosed before 2013 were excluded from this cohort. Only patients fulfilling the 2012 EULAR/ACR classification criteria were included in our cohort.

In summer 2017, all GPs in our catchment area were informed about the FTC’s establishment, including a three-hour educational meeting about the PMR/GCA challenges and the FTC approach. For better service of the local GPs’ or other medical departments’ needs, direct telephone access to a rheumatologist was introduced. A rheumatologist evaluated all referrals to our outpatient clinic, and in case of PMR suspicion, the patient was examined within 3 days. Furthermore, patients admitted to the emergency medical department with PMR symptoms/suspicion could be discharged and given an appointment in the FTC within 24 h if the referring GP finds it appropriate. Detailed patient history and clinical examination were performed at the FTC, including musculoskeletal (MSK) and vascular ultrasound (US).

REDCap (Research Electronic Data Capture) [17] tools hosted at OPEN Odense University were used for data collection and management. The study was approved by the Danish Health Authority and the Data Agency of the South-West Hospital in Esbjerg.

The statistical analysis was performed by using STATA Ver. 12 SE. The patient cohort included all patients registered with a diagnosis of PMR in the Department of Rheumatology in South-West Jutland Hospital from April 2013 to December 2018. Patients admitted to the medical emergency department with an inpatient care duration of > 8 h were defined as hospitalised. For comparisons between groups, the independent samples t-test was used for continuous variables. For the categorical variables, the Chi-square test or Fisher’s exact test was used when appropriate. *P* values < 0.05 were considered statistically significant.

## Results

**Fig. 1:**
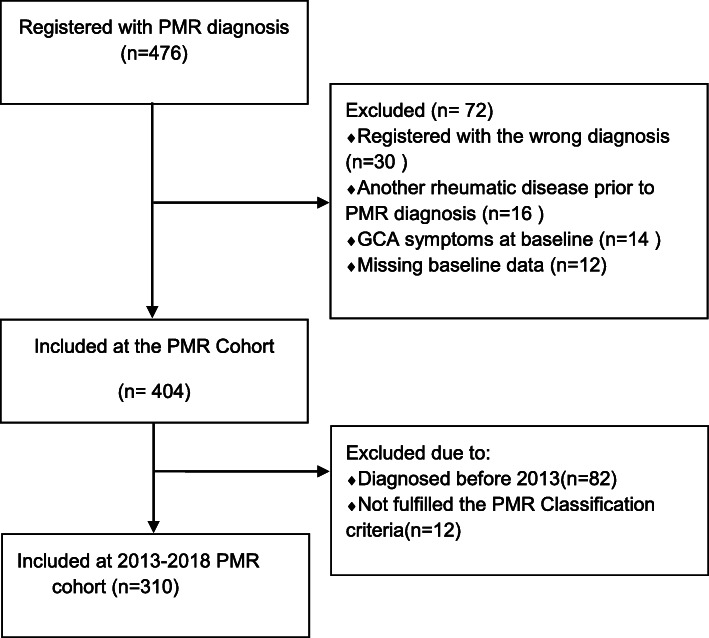
Flowchart for polymyalgia rheumatica cohort. PMR: Polymyalgia rheumatica, GCA: Giant cell arteritis

Four hundred seventy-six patients were registered with the diagnosis of PMR during the study period. Seventy-two patients were excluded from the study: 30 due to incorrect diagnosis, 16 suffered from other rheumatic diseases before the PMR diagnosis, 12 patient records were incomplete, and 14 had concomitant GCA symptoms at baseline. Furthermore, 82 of the identified patients were excluded due to the time of diagnosis before 2013. To summarise, in a period of 6 years (2013–2018), 324 patients were diagnosed with PMR, where 310 of them fulfilled the 2012 EULAR/ACR classification criteria and were included in the study (Fig. 1).

During the study period, 54 patients were admitted to the South-West Jutland Hospital with a mean duration of 4.02 inpatient days of care. In 2013, data were collected only from April to December, and the annual number of newly diagnosed PMR patients was 31, of whom seven were hospitalised. In 4 years (2014–2017), the annual number of newly diagnosed PMR patients was relatively stable, with a mean value of 59 patients (range 52–68). The hospitalisations rates were equal for 2014 (*n* = 12), 2015 (*n* = 13), 2016 (*n* = 12), and slightly decreased at 2017 (*n* = 8).

The baseline characteristics of all the patients are presented in Table 1. There were no differences among hospitalised patients compared to non-hospitalised patients regarding gender and PMR-related symptoms. Hospitalised patients were more likely to have higher initial CRP (*p* <  0.0001), shorter duration of symptoms (*p* = 0.0018); they were slightly older (mean 74.11 vs 71.12 years) and with a slightly higher initial prednisolone dose (mean 20.75 vs 18.01 mg) compared to non-hospitalised patients (Table 1).

**Table 1:** Baseline characteristics of all patients and comparison between hospitalised vs non-hospitalised patient groups

	All Patients N 310	Group A Hospitalised N 54	Group B Non-Hospitalised N 256	Group A vs B *P* values
Age (years) mean ± SD	71.63 ± 7.79	74.11 ± 8.24	71.12 ± 7.61	0.010
Gender-female(%)	56.1%%	56.6%	56.0%	1
Duration of symptoms before the admission (weeks) mean ± SD	11.36 ± 11.54	6.94 ± 5.60	12.37 ± 12.30	0.001
CRP (mg/lt) mean ± SD	55.37 ± 46.50	99.53 ± 59.36	45.82 ± 36.96	< 0.00001
Morning stiffness duration > 45 min	94.8%	98.1%	94.1%	0.220
Hip pain or limited range of motion	71.3%%	78.8%	69.8%	0.182
RF and ACPA negative	96.1%%	98.1%	95.7%	0.675
Absence of other joint involvement	71.9%	71.7%	72.0%	1
Initial prednisolone dose(mg) mean ± SD	18.49 ± 8.50	20.75 ± 10.02	18.01 ± 8.08	0.030

*SD* Standard deviation, *CRP* C-reactive protein, *RF* Rheumatoid factor, *Anti-CCP* Anti-cyclic citrullinated peptide

**Fig. 2:**
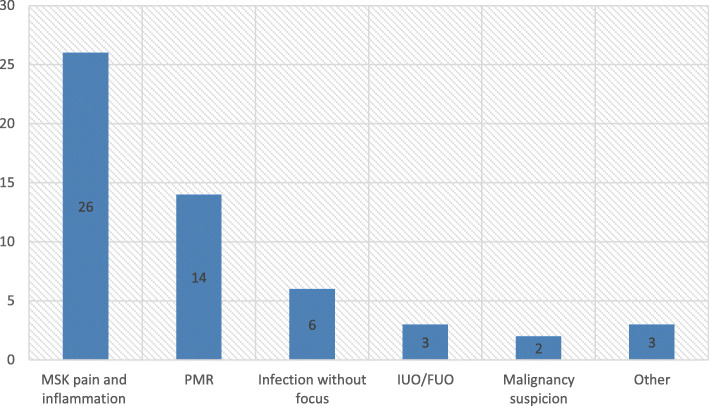
Initial reasons for hospitalisation. MSK: musculoskeletal; PMR: Polymyalgia rheumatica; IUO/FUO: Inflammation/Fever of Unknown Origin

In 48% of the hospitalised patients, the reason for the initial referral to the emergency medical department was musculoskeletal pain and abnormal inflammatory markers, followed by tentative PMR diagnosis (25%). Other initial tentative diagnoses included infection (*n* = 6), fever or inflammation of unknown origin (*n* = 3), and malignancy suspicion (*n* = 2) (Fig. 2). The majority of the hospitalised patients (70%) were treated initially with moderate doses (15-25 mg) of glucocorticosteroids (GS) followed by antibiotics (18%) (Supplementary Fig. S1).

During hospitalisation, various diagnostic imaging procedures were performed to exclude underlying conditions other than PMR. The use of imaging modalities (computer tomography scanning of chest and abdomen, chest X-ray, abdominal ultrasound, magnetic resonance of the spine) was significantly higher compared to the non-hospitalised group (81,5% vs 15,9%, *p* <  0,00001) (Table 2). No pathological findings other than these attributed to PMR on PET-CT and bone scintigraphy were observed on the above examinations. The most common imaging modality performed in PMR patients was the musculoskeletal US, which was carried out in 64 and 63.3% of hospitalised and non-hospitalised patients, respectively. The most common findings were inflammatory changes of the shoulders/hips (82% in hospitalised patients versus 78% in non-hospitalised patients).

**Table 2:** Imaging modalities used other than musculoskeletal ultrasound between the group of hospitalised and non-hospitalised patients

	Group A Hospitalised N 54	Group B Non-Hospitalised N 256	*P* values
Imagining examination was performed n (%)	44(81.4%)	37(14.3%)	< 0,00001
PET-CT n(%)	3(5.5%)	9(3.5%)	0.444
CT-Chest/Abdomen n(%)	18(33.3%)	10(3.8%)	< 0.00001
Chest X-ray n(%)	18(33.3%)	5(1.9%)	< 0.00001
Bone Scintigraphy n(%)	3(5.5%)	4(1.5%)	0.156
Abdominal US n(%)	6(11.1%)	2(0.7%)	0.0005
Axial MR n(%)	5(9.2%)	6(2.3%)	0.027

*PET-CT* Positron emission tomography-computed tomography, *CT* Computer tomography, *US* Ultrasound, *MR* Magnetic resonance

After implementing the FTC in January 2018, the hospitalisation rates decreased significantly from 20.4 to 3.5% (*p* = 0.001) and the inpatient days of care from a mean of 4.15 days to 1 day (*P* <  0.00001). In two cases, the referral to the emergency department was converted to an FTC appointment the day after; both patients’ final diagnosis was PMR. No differences regarding clinical and laboratory characteristics between the two groups were observed (Table 3). The symptoms’ duration before diagnosis was as expected, significantly (*p* = 0.001) decreased in the FTC group (Table 3). No cases with admission to the medical emergency department and a short duration of stay (≤8 h) before discharging were observed, both in the 2013–2017 and 2018 cohort.

**Table 3:** Baseline characteristics between the patient cohorts before and after implementation of the FTC

	Diagnosed during the period 2013–2017	Diagnosed after the implementation of FTC	*P* values
Number of patients	254	56	
Age (years) mean ± SD	71.60 ± 8.04	71.79 ± 6.6	0.861
Gender (female)	57.9%%	48.2%	0.233
Duration of symptoms (weeks) mean ± SD	12.30 ± 12.30	6.79 ± .72	0.001
CRP (mg/lt) mean ± SD	55.10 ± 48.41	56,5 ± 37.2	0.830
Morning stiffness	94.9%	92.7%	0.527
Hip pain or limited range of motion	73.4%	61.8%	0.149
RF and anti-CCP negative	95.3%	100%	0.133
Absence of other joint involvement	71.3%	75%	0.627
Initial prednisolone dose (mg) mean ± SD	18.37 ± 8.33	19,2 ± 9.25	0.550
Hospitalised n(%)	52 (20.4%)	2 (3.5%)	0.001
Inpatient days of care mean ± SD	4.15 ± 3.1	1 ± 0	< 0.00001

*FTC* Fast track clinic, *SD* Standard deviation, *CRP* C-reactive protein, *RF* Rheumatoid factor, *Anti-CCP* Anti-cyclic citrullinated peptide

According to the Danish Ministry of Finance, the healthcare costs (inpatient and outpatient) in the Danish hospitals are calculated using the DRG system [18]. The annual cost for 56 new diagnosed patients was reduced by 65% after introducing the FTC in 2018 compared to the 2013 to 2017 period.

## Discussion

To our knowledge, this is the first study investigating the hospitalisation rates among newly diagnosed patients with PMR and the reasons for hospitalisation. In our study cohort, one in five newly diagnosed PMR patients was referred for hospitalisation at disease presentation before establishing the FTC. Our results demonstrated a significant difference (< 0.0001) on the initial CRP level between hospitalised and non-hospitalised patients (mean ± standard deviation-SD) 99.53 ± 59.36 vs 45.82 ± 36.96 mg/lt, without differences in the PMR-related symptoms. The high initial CRP levels and the consequent fear of a potentially severe underlying disease may be the main reason for admission to the hospital (Fig. 2).

In a Swedish study from 1995, which investigated the mortality among 220, temporal biopsy negative PMR patients [11] 72% of all PMR patients were hospitalised. However, in this study, the patients with initial GCA-related symptoms were not excluded, leading to a potential selection bias. Furthermore, inpatient days were not presented, and it is generally unclear if the hospitalisation included patients referred from the rheumatology outpatient clinic. A Norwegian study from 1997 analysed the incidence of GCA/PMR in the county of Aust Agder (catchment area of 98,000 inhabitants) over 8 years [7]. They encouraged GPs to refer patients with GCA/PMR suspicion to the local Department of Rheumatology. During the study period, only three patients were referred to the department of internal medicine with a tentative PMR diagnosis, while in the previous 8 years, 31 cases of PMR & GCA were diagnosed by other than Rheumatology departments. However, it is unclear if these patients were hospitalised or diagnosed in the outpatient clinic.

In a short communication a research group from Turkey reported, that about 30% of the PMR patients were hospitalised for a mean period of 7 ± 3 days before referral to rheumatology unit [12]. Somehow, in this study, initial GCA related symptoms as headache and sight loss were reported in 43 and 7.5% of the patients. Furthermore, no information is available about hospitalisations reasons and when during the disease patients were hospitalised. Another significant difference was the very high duration (13.2 ± 12.4 months) between the first symptom onset and admission or referral to a rheumatology clinic, compared to our 2013–2017 cohort (mean 12.30 ± 12.30 weeks). An explanation can be that the physician might not be so aware and experienced on PMR diagnosis in countries with low PMR incidences [2].

In our study, the main reason for hospitalisation was musculoskeletal symptoms in association with significantly elevated levels of inflammatory markers. Non-rheumatologists initially evaluated hospitalised patients in the emergency medical department, which could be the reason for the frequent initiation of treatment with antibiotics (Supplementary S1), a high number of inpatient days of care (Table 3), higher initial prednisolone dose (Table 1), and finally increased use of imaging modalities (Table 2) to exclude other conditions, compared to the non-hospitalised group. In the study by Schönau V et al. [19], patients with fever or inflammation of unknown origin (FUO and IUO) were referred to the Immunology and Infectious Disease Clinic for further examinations. With the help of a PET-CT scanning and later on confirmed by a rheumatologist, the diagnosis of PMR was made in 6% of patients with FUO and 18% with IUO. These results are in line with our observations that patients with PMR symptoms can be referred to a non-rheumatological department or hospitalised, leading to a diagnostic delay and excessive, expensive examinations.

Hospitalised patients had a significantly (*p* = 0.0018) shorter duration of symptoms before diagnosis compared to the non-hospitalised group. In 2018, using the FTC approach, the duration of symptoms before diagnosis decreased significantly and was similar to hospitalised patients. The difference in symptoms’ duration can be explained by the time from referral to evaluation in the rheumatological outpatient clinic (approximately 4–6 weeks) before establishing the FTC.

Twelve patients with a PMR diagnosis made by a rheumatologist did not fulfil the classification criteria for PMR. In 10 of the cases, the reason was initially normal inflammatory markers. ESR and CRP usually support PMR diagnosis, and increased inflammatory markers are mandatory for satisfying the PMR classification criteria algorithm [16]. Studies, however, show that 1.5–22.2% of patients with PMR present with normal acute-phase reactants [20, 21]. In our study, none of the 12 excluded patients was hospitalised.

The fact that PMR is the second most common rheumatic disease in the elderly [22, 23] and the number of people aged over 60 years expected to increase dramatically in all countries with a high prevalence of PMR [24] emphasises the importance of our findings. The rapid evaluation of patients with PMR by using the FTC approach led to a significant decrease in hospitalisation rates and the inpatients days of care, highlighting another advantage of the FTC as a potentially more cost-effective approach for diagnosing PMR.

One of this study’s strengths is that it performed in one endemic region for PMR [2, 3], where GPs are used to managing patients with these conditions [3]. The electronic journal system (Cambio COSMIC) ensured all medical records’ availability, hence collecting all necessary clinical and paraclinical data. Besides, the emergency department in SVS is the only available department for admissions in our catchment area, securing the registration of hospitalised PMR patients.

Our study has limitations. The study’s design is retrospective and included only patients referred to the hospital, thereby excluding patients diagnosed and treated in primary care. Nevertheless, it is a common international practice, GPs treating PMR patients and referring challenging cases to specialist healthcare for advanced diagnostics and treatment. Consequently, our study reflects the real population of PMR patients referred to specialist healthcare. The groups of hospitalised vs non-hospitalised patients were comparable in PMR related symptoms, but we could not compare the severity of symptoms, disability, or comorbidities, which may be one of the reasons for hospitalisation. Somehow, diagnostic imaging modalities were performed in the majority of hospital patient (Table 2) and other causes for PMR symptoms as malignancy or infections were excluded. Furthermore, none of the patients in the 2018 cohort developed malignancy during the first year of follow-up. The association between PMR and cancer is unclear, with previous studies yielding mixed results [11, 25–28].

Another limitation is that we did not routinely examine the 2013–2017 PMR cohort for subclinical GCA. It is reported in 16–21% of PMR patients, and the possibility of coexistent GCA arises in PMR patients with constitutional symptoms and markedly elevated acute phase reactants ([29]). In our cohort, the hospitalised patients underwent a CT scan of chest/abdomen with contrast or PET-CT in almost 40% of cases (Table 2) without evidence of large-vessel vasculitis. Furthermore, none of the hospitalised patients has was diagnosed with GCA during the first-year follow-up after the treatment initiation. Another limitation of our study is that we included patients with an established PMR diagnosis but not patients with PMR suspicion ending with other final diagnoses. We identified our patient population using an electronic database, where patients are registered according to their final diagnosis and not the tentative/referred diagnosis. More studies evaluating the impact of FTC on patients with PMR suspicion are warranted in the future.

## Conclusion

This is the first study to investigate the hospitalisation rates and the reason for hospital admissions of newly diagnosed PMR patients. Furthermore, the implementation of an FTC approach decreases the hospitalisation rates and inpatients’ days of care. Future research should focus on PMR diagnosing challenges and how the primary sector and hospital departments can optimise their collaboration.

## Supplementary Information

**Additional file 1: Figure S1.** Initial treatment during hospitalization.

## Data Availability

The data that support the findings of this study are not publicly available, and restrictions apply to the availability of these data according to the Danish Data Protection Regulation. Data are, however, available from the authors upon reasonable request and with permission of the South-West Hospital in Esbjerg, legal services of the Research & Innovation Organisation, and approval from the Danish Data Protection Agency.
