# Supplementary material for: The fast-track outpatient clinic significantly decreases hospitalisation rates among polymyalgia rheumatica patients
Source: BMC Rheumatol. 2021 Oct 5;5:37. doi: 10.1186/s41927-021-00210-6 (PMC8491370; doi:10.1186/s41927-021-00210-6)
Supplement: Supplementary file 1 — Additional file 1: Figure S1. Initial treatment during hospitalization. [file 41927_2021_210_MOESM1_ESM.docx]

Supplementary figure S1. Initial treatment during hospitalization

GS: Glucocosticosteroids
